# Author’s reply to ‘Rickettsia retinitis cases in India: a few comments’

**DOI:** 10.1186/s12348-016-0086-z

**Published:** 2016-06-07

**Authors:** Ankush A. Kawali, Padmamalini Mahendradas, Kanav Gupta, Priya Srinivasan, Kavitha Avadhani, Naresh Kumar Yadav, Rohit Shetty

**Affiliations:** Uveitis and Ocular Immunology Services, Narayana Nethralaya, Bangalore, India; Vitreo-retinal Services, Narayana Nethralaya, Bangalore, India; Cornea and Refractive Services, Narayana Nethralaya, Bangalore, India

**Keywords:** Weil-Felix test, Optical coherence tomography, Rickettsial retinitis, Antibiotics, Steroids

## Abstract

Diagnosis of rickettsial retinitis remains presumptive when gold standard tests are not available or not done due to financial constrains. History of tick bite followed by fever with skin rash particularly in winter and spring season may point towards Rickettsiosis. The absence of scarring post resolution of rickettsial retinitis suggests inner retinal involvement in contrast to toxoplasmosis. Bilaterality of the disease, 2–4 weeks of latent period, and multifocal nature of retinitis lesions (cotton wool spot-like lesions) especially around the disc and posterior pole may suggest an immune response to recent systemic infection. The use of only antibiotics or only steroids or both together for treatment of rickettsial retinitis is controversial and warrants randomized controlled trials.

Editor,

We thank Kaushik Tripathi, Rohan Chawla and co-authors for their interest in our article and giving us the opportunity to re-look and clarify a few points from our work [1]. We would like to reply to their comments in the following manner:

1. For our study, Weil-Felix test (WFT) was performed using the commercially available kit (Plasmatech, UK) as per the manufacturer’s instructions which gives a positive result to agglutination titres of ≥160 [2]. We are completely aware that WFT is not a gold standard test to diagnose rickettsial diseases and hence, we used a term “presumed rickettsial retinitis” in the report.
  Multifocal retinitis post febrile illness in Indian scenario is commonly due to chikungunya, dengue, West Nile virus (WNV) and rickettsial infections. Ruling out other causes (chikungunya, dengue, WNV), taking appropriate history of tick bite rather than mosquito bite and skin rashes will increase suspicion for rickettsial retinitis. Of late, we processed stored serum sample of our patients of “presumed rickettsial retinitis” for WNV serology and PCR which showed negative results. Although WFT is not a gold standard test, it helps to subclassify rickettsial diseases (Indian tick typhus, epidemic typhus and scrub typhus) whereas more definite tests like IgG and IgM enzyme-linked immunosorbent assay (ELISA) and PCR need to be carried out separately for each rickettsial organism and hence the cost.
2. OCT findings in toxoplasmosis are well described by Goldenberg et al. who studied 17 active lesions in 11 eyes which showed full-thickness retinal involvement in all [3], although exception to this can occur, especially in early presentation of toxoplasma retinitis. The absence of scar formation or absence of significant RPE changes after resolution of rickettsial retinitis lesions may suggest inner retinal involvement in contrast to toxoplasma retinitis. Rickettsial retinitis lesions are almost always multiple in numbers and appear around the disc and at the posterior pole. They are very much similar to cotton wool spots (CWS) or in fact could be CWS only (as described in noninfectious HIV retinopathy [4]). The presence of vitritis and gross macular oedema encourages calling them retinitis lesions rather than CWS. Isolation of all possible rickettsial organisms from ocular fluid was not possible in our study, but aqueous sample for PCR scrub typhus was negative in three cases.
3. Whether to continue or to restart the antibiotic treatment for rickettsial retinitis, when patients have already been treated by their physician for the fever (generally 2–4 weeks prior to presentation), is debatable and warrants randomized control trials. As we mentioned, bilaterality of retinitis lesions in 9 out of 10 immunocompetent patients, an interval of 2 to 4 weeks between the systemic and ocular presentation and CWS-like retinitis lesions around the disc points towards an immune-driven process. Whether one can detect active replicating rickettsial organisms in these retinitis lesions or not is questionable. The possibility of a para-infectious process is more likely. Response to steroid-antibiotics versus only steroids needs to be evaluated in further studies. We hesitate to give any strong treatment recommendation based on our report. Out of other three cases of ‘steroid only’ treatment in our series, one was a pregnant lady and the other two were already on systemic steroids started elsewhere and were showing favourable response.
4. Kyrieleis plaques as described in toxoplasmosis and acute retinal necrosis were not seen by us in the reported case series; rather, we noticed an occluded arteriole on FFA in one of our patient (as shown in Figure 2 of the article) [1]. Since the inclusion criterion for this study was retinitis, it is possible that we would have missed cases of rickettsial vasculitis without retinitis. But recently, a new case of presumed rickettsial retinitis did show presence of Kyrieleis plaques which was not included in the report.

## Abbreviations

SD-OCT: spectral domain optical coherence tomography
WFT: Weil-Felix test
WNV: West Nile virus
